# GDF11 alleviates neointimal hyperplasia in a rat model of artery injury by regulating endothelial NLRP3 inflammasome activation and rapid re-endothelialization

**DOI:** 10.1186/s12967-022-03229-6

**Published:** 2022-01-15

**Authors:** Lei Li, Yan Gao, Zhenchuan Liu, Chenglai Dong, Wenli Wang, Kaiqin Wu, Shaorui Gu, Yongxin Zhou

**Affiliations:** 1 Department of Thoracic and Cardiovascular Surgery, Tongji Hospital, School of Medicine, Tongji University, Shanghai, 200065 China; 2 Department of Respiratory and Critical Care Medicine, Huai’an Second People’s Hospital and The Affiliated Huai’an Hospital of Xuzhou Medical University, Huai’an, 223001 China

**Keywords:** Growth differentiation factor 11, Re-endothelialization, NLRP3 inflammasome, LOX-1, Neointima formation

## Abstract

**Background:**

Neointimal hyperplasia induced by interventional surgery can lead to progressive obliteration of the vascular lumen, which has become a major factor affecting prognosis. The rate of re-endothelialization is known to be inversely related to neointima formation. Growth differentiation factor 11 (GDF11) is a secreted protein with anti-inflammatory, antioxidant, and antiaging properties. Recent reports have indicated that GDF11 can improve vascular remodeling by maintaining the differentiated phenotypes of vascular smooth muscle cells. However, it is not known whether and how GDF11 promotes re-endothelialization in vascular injury. The present study was performed to clarify the influence of GDF11 on re-endothelialization after vascular injury.

**Methods:**

An adult Sprague–Dawley rat model of common carotid artery balloon dilatation injury was surgically established. A recombinant adenovirus carrying GDF11 was delivered into the common carotid artery to overexpress GDF11. Vascular re-endothelialization and neointima formation were assessed in harvested carotid arteries through histomolecular analysis. CCK-8 analysis, LDH release and Western blotting were performed to investigate the effects of GDF11 on endothelial NLRP3 inflammasome activation and relevant signaling pathways in vitro.

**Results:**

GDF11 significantly enhanced re-endothelialization and reduced neointima formation in rats with balloon-dilatation injury by suppressing the activation of the NLRP3 inflammasome. Administration of an endoplasmic reticulum stress (ER stress) inhibitor, 4PBA, attenuated endothelial NLRP3 inflammasome activation induced by lysophosphatidylcholine. In addition, upregulation of LOX-1 expression involved elevated ER stress and could result in endothelial NLRP3 inflammasome activation. Moreover, GDF11 significantly inhibited NLRP3 inflammasome-mediated endothelial cell pyroptosis by negatively regulating LOX-1-dependent ER stress.

**Conclusions:**

We conclude that GDF11 improves re-endothelialization and can attenuate vascular remodeling by reducing endothelial NLRP3 inflammasome activation. These findings shed light on new treatment strategies to promote re-endothelialization based on GDF11 as a future target.

**Supplementary Information:**

The online version contains supplementary material available at 10.1186/s12967-022-03229-6.

## Background

Cardiovascular disease is still the main cause of human death. The rupture and shedding of atherosclerotic plaques is an important factor related to mortality in acute coronary artery disease [1]. Percutaneous intracavitary interventional therapy is minimally invasive and has a definite curative effect. This procedure is currently an important treatment technique for cardiovascular diseases and is widely used in clinics. However, intimal neoplasia induced by interventional surgery can lead to restenosis, which has become a major factor affecting prognosis [2]. The rate of re-endothelialization is inversely related to neointima formation [3]. Therefore, promoting the re-endothelialization of injured blood vessels is urgently needed to reduce delayed restenosis and improve patients’ quality of life after interventional surgery.

Growth differentiation factor 11 (GDF11) belongs to the transforming growth factor-β superfamily and is involved in the regulation of a variety of important biological functions. As a secreted protein, GDF11 has anti-inflammatory, antioxidant and antiaging properties [4, 5]. Studies have confirmed that higher levels of GDF11 are associated with lower risks of cardiovascular events and death, and this molecule is a potential new target for improving cardiovascular outcomes [6]. Recent studies have found that GDF11 can maintain the contractile phenotype of vascular smooth muscle cells and inhibit the development of thoracic aortic dissection and sclerosing arterial disease [7, 8]. Moreover, previously published data indicate that GDF11 may regulate atherosclerosis by affecting the function of endothelial cells [9]. The functional status of endothelial cells is closely related to the formation of neointima. However, why GDF11 regulates endothelial function to inhibit neointima formation has not yet been fully elucidated.

The NLRP3 inflammasome is an intracellular innate immune receptor that recognizes a diverse range of stimuli. NLRP3 activation causes the assembly of a large multiprotein complex known as the NLRP3 inflammasome and leads to the activation of caspase-1, which subsequently produces bioactive IL-1β. Moreover, active caspase-1 induced by the NLRP3 inflammasome can induce other nonclassical effects, such as cell pyroptosis and high cell permeability [10]. The NLRP3 inflammasome pathway is implicated in vascular neointima hyperplasia [11]. It has been confirmed that endothelial NLRP3 inflammasome activation contributes to the development of neointima formation [10, 12, 13]. NLRP3 is one of the GDF11 target genes confirmed by previous experiments [14, 15]. However, whether GDF11 can attenuate vascular remodeling by targeting the endothelial NLRP3 inflammasome signaling pathway remains largely unknown.

Bioactive lipids are important signaling molecules in many physiological processes [16]. Many studies have shown that the abnormal synthesis and distribution of these lipids are related to many diseases, especially in the cardiovascular system [17]. Lysophosphatidylcholine (lysoPC), a bioactive phospholipid generated by various biological processes, is abundant in plasma and accumulates in damaged blood vessels [18, 19]. Previous studies have demonstrated that its inhibition of vascular endothelial cell function is unfavorable for the restoration of endothelial integrity after injury [20, 21]. Moreover, studies have shown that lysoPC is associated with the activation of endothelial NLRP3 inflammasomes [22, 23].

In the present study, we explored the mechanism through which GDF11 promotes the repair of damaged blood vessels. Our research found that GDF11 improved re-endothelialization and regulated the lysoPC-induced activation of the endothelial NLRP3 inflammasome by inhibiting LOX-1-dependent endoplasmic reticulum stress (ER stress), which provides an important theoretical basis for GDF11 inhibiting vascular neointima hyperplasia and promoting the repair of damaged blood vessels.

## Materials and methods

### Cell cultures

Human umbilical vein endothelial cells (HUVECs), a human endothelial cell lineage, were obtained from the American Type Culture Collection (Rockville, MD, USA) and maintained in DMEM containing 10% FBS and 1% penicillin/streptomycin in a humidified atmosphere of 5% CO_2_ at 37 °C. LysoPC (Sigma Aldrich, MS, USA) was dissolved in PBS and preserved at – 20 °C. When the cells reached approximately 80% confluence, they were treated with lysoPC for 24 h. Cells in the GDF11 and inhibitor groups were pretreated with GDF11 (50 ng/mL) and 4-PBA (5 mM) for 1 h prior to exposure to 50 ug/mL lysoPC for another 24 h.

### Cell viability assay

Cell viability was evaluated using a CCK-8 (Dojindo, Kumamoto, Japan). Briefly, cells were seeded in 96-well plates at a density of 1 × 10^4^ cells/well overnight. After treatment for 24 h as described in the Cell cultures section, 10 μL of CCK-8 solution was added to the culture medium in each well, and the cells were then incubated in humidified 95% air and 5% CO_2_ for 1 h at 37 °C. The absorbance at a wavelength of 450 nm was measured using a microplate reader (Thermo Fisher Scientific, NYC, USA).

### Determination of the LDH activity

Cell incubation was carried out as previously described in the Cell cultures section, after which cytotoxicity was determined by monitoring LDH release with an LDH cytotoxicity assay kit (Beyotime, Shanghai, China). Briefly, after the predetermined period of time, the cell culture plate was centrifuged at 400*g* for 5 min. We took 120 μL of supernatant from each well and added it to the corresponding well of a new 96-well plate and then added 60 μL of LDH detection solution to each well. This plate was incubated at room temperature for 30 min, after which the absorbance at 490 nm was measured using a microplate reader (Thermo Fisher Scientific, NYC, USA).

### Hoechst 33342 and propidium iodide (PI) staining

HUVECs were seeded in 6-well plates at a density of 2 × 10^5^ cells/well. After treatment, the cells in each group were washed with PBS 3 times and stained with 5 μL of Hoechst 33342 and 5 μL of PI (Beyotime, Shanghai, China) for 20 min at 4 ℃. The stained cells were observed under a fluorescence microscope (Olympus, Tokyo, Japan) and evaluated using the ImageJ software.

### Western blot analysis

As usual, HUVECs were seeded in 6-well plates at a density of 2 × 10^5^ cells/well. After treatment, the HUVECs were analyzed using Western blotting [24]. Briefly, the cells were lysed with RIPA buffer containing 1% proteinase inhibitor, PMSF, at 4 ℃. Then, the samples underwent centrifugation for 10 min at 4 ℃, the supernatant was collected, and the protein concentration was measured with a BCA kit (Beyotime, Shanghai, China). The protein samples were separated by SDS–polyacrylamide gel electrophoresis and transferred onto a poly(vinylidene fluoride) (PVDF) membrane. After the membrane was blocked with 5% nonfat milk for 1 h at room temperature, it was incubated with primary antibodies against CHOP (Beyotime, Shanghai, China), Casp1 p20 (Cell Signaling Technology, MA, USA), GDF11, LOX-1, NLRP3, IL-1β, GSDMD-N, or GRP78 (Abcam, Cambridge, UK) at 4 °C overnight. This step was followed by incubation with a secondary antibody for 1 h at 37 °C. The proteins were scanned using the Odyssey Imaging System, and the band intensities were quantified using ImageJ. GAPDH (Abcam, Cambridge, UK) was used as an internal control. The protein expression levels of NLRP3, Casp1 p20, and IL-1β in the harvested artery specimens were also determined using Western blotting.

### RNA extraction and qPCR

Total RNA was extracted from HUVECs using TRIzol reagent (Invitrogen, CA, USA) following the manufacturer’s instructions. The concentration of RNA was measured using a NanoDrop (Thermo Fisher Scientific, NYC, USA). Reverse transcription and qPCR were performed as described previously [25]. To carry out mRNA quantification, we used a reverse transcription kit (Vazyme, Nanjing, China) for the reverse transcription of total RNA according to the manufacturer’s instructions. Then, the SYBR Green Master Mix (Vazyme, Nanjing, China) was utilized to perform qPCR, and a Roche Light Cycler system (Roche, Switzerland) was used for analysis. The sequences of the primers used were as follows: LOX-1, F: 5′-GAGTGAACATATCCATCATC-3′, R: 5′-TGGAGACATATGAATCTCAA-3′; GAPDH, F: 5′-AAGAAGGTGGTGAAGCAGGC-3′, R: 5′-TCCACCACCCAGTTGCTGTA-3′.

### Small-interfering RNA transfection

Small-interfering RNA against LOX-1 (si-LOX-1) was synthesized by RiboBio Biotechnology Company (Guangzhou, China). For transfection, 50 nM si-LOX-1 and scrambled siRNA were delivered into cells using the Lipofectamine™ 2000 transfection reagent (Invitrogen, CA, USA) according to the manufacturer’s instructions. HUVECs were harvested for other experiments 48 h after transfection.

### Animal model and adenovirus transduction

Animals were treated in accordance with the Guide for the Care and Use of Laboratory Animals published by the US National Institutes of Health, and this experiment was approved by the Animal Ethics Committee of the Tongji University School of Medicine. Male Sprague–Dawley rats (weight: 300–350 g) were anesthetized by intraperitoneal injection of pentobarbital solution (30 mg/kg) and then injected with heparin (100 U/kg) in the tail vein. The procedure for the rat carotid artery balloon injury model has been described previously [26]. In brief, a 2F balloon catheter (Fogarty, E-060-2F, Baxter) was inserted via the right external carotid artery into the common carotid artery, and the balloon was inflated with saline and drawn toward the arteriotomy site three times to ensure complete intimal injury. The injured artery was washed with PBS. Then, an adenovirus encoding GDF11 or control GFP (1 × 10^9^ pfu, Genechem Co., Ltd, Shanghai, China) was injected into the balloon-injured rat carotid arteries via the incision of the right external carotid artery and kept in situ for 30 min. The solution was subsequently withdrawn from the infected area, and blood flow was restored. For determination of the efficiency of adenovirus delivery into the carotid artery, carotid arteries from the Ad-GFP-infected rats were observed under fluorescence microscopy (Olympus, Tokyo, Japan).

### Evans blue staining

To measure the re-endothelialization area 7 days after vascular injury, we injected 5% Evans blue dye (Sigma Aldrich, MS, USA) via the tail vein. After 30 min, arteries were dissected from the carotid bifurcation and opened longitudinally to analyze the denuded and recovered areas. Images were obtained using a digital camera (Cannon, Japan). Re-endothelialization was measured using image analyzer software (Image-Pro Plus 6.0).

### Immunohistochemistry

After the rats were sacrificed, the injured aortic tissues were fixed with 4% paraformaldehyde solution, embedded in paraffin, and cut into 6 μm sections, which were prepared and incubated with rabbit anti-CD31 (Servicebio, China) overnight at 4 °C. After routine immunohistochemistry, the sections were observed under a microscope (Olympus, Tokyo, Japan). Image-Pro Plus 6.0 software was used to calculate the ratio of CD31-expressing cells to the total number of cells in the arterial lumen wall to measure the re-endothelialization rate for each cross-section.

### Hematoxylin and eosin (HE) staining

Paraffin sections of 6 μm were deparaffinized with xylene and hydrated with ethanol. The sections were dyed with hematoxylin and then stained in eosin dye solution. Images were obtained with an inverted phase contrast microscope (Olympus, Tokyo, Japan). Neointima formation (intimal, medial, and intimal/medial areas) was measured using Image-Pro Plus 6.0 image analysis software.

### Statistical analysis

All the data are expressed as the means ± SEMs, and all the experiments were performed in triplicate. Comparisons between groups were performed using unpaired Student’s t tests. Analysis of variance (ANOVA) followed by Tukey’s multiple comparison test was utilized to test for differences among groups. The results were analyzed using GraphPad Prism 9 (GraphPad Software, Inc., USA). P < 0.05 was regarded as significant.

## Results

### GDF11 inhibited NLRP3 inflammasome activation and pyroptosis in lysoPC-treated human endothelial cells

**Fig. 1:**
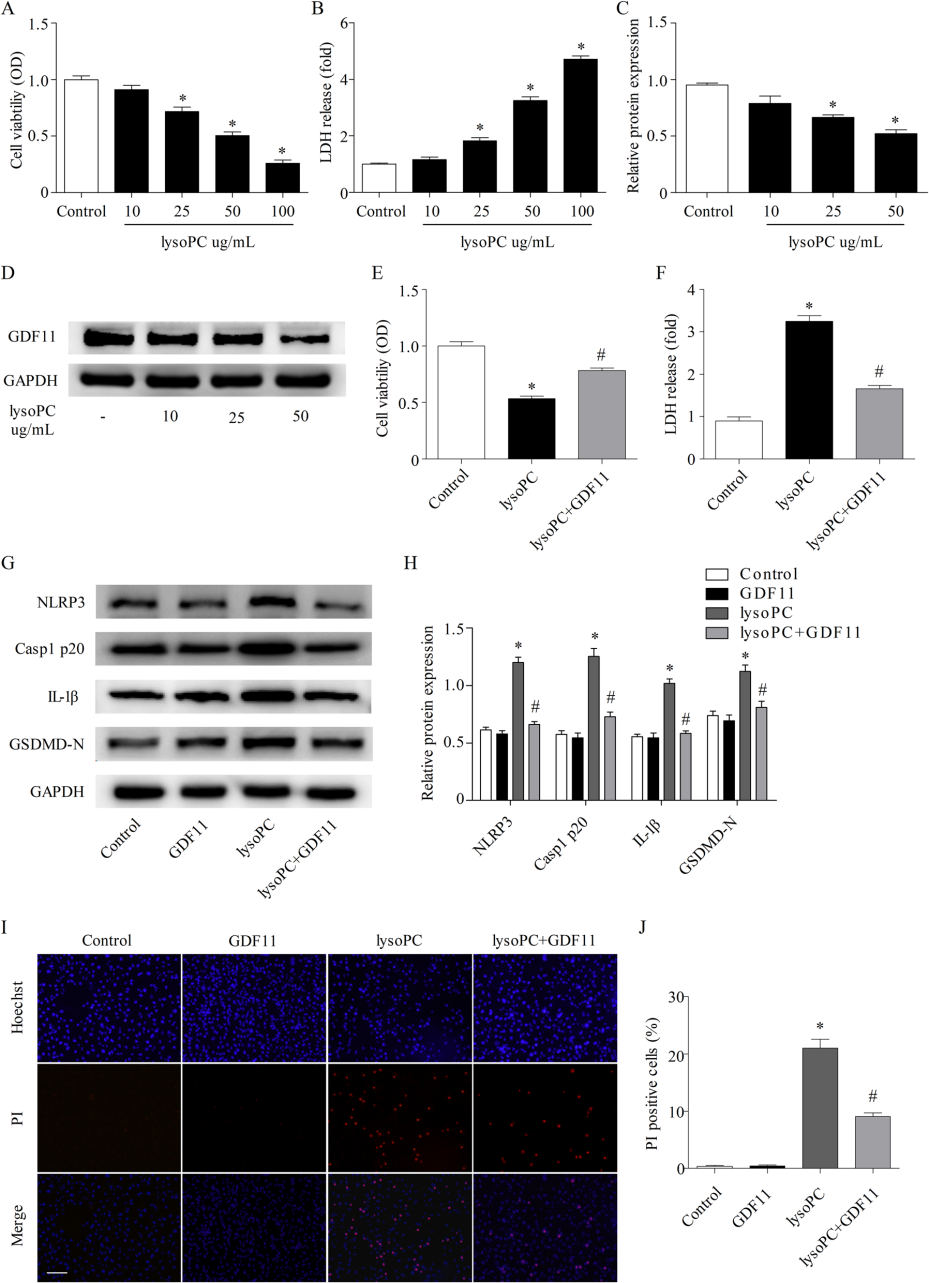
GDF11 inhibited NLRP3 inflammasome activation and pyroptosis in the lysoPC-treated human endothelial cells. **A** Cell viability was detected by CCK-8 assays after treatment with different concentrations of lysoPC for 24 h. **B** Cytotoxicity was evaluated by LDH release. **C**, **D** The effect of lysoPC on the expression of GDF11 in human endothelial cells was detected by Western blotting. **E** Cells were pretreated with 50 ng/mL GDF11 for 1 h, followed by lysoPC treatment for 24 h. Cell viability was detected by CCK-8 assays. **F** Cytotoxicity was evaluated by the LDH release. **G**, **H** The effect of GDF11 on the expression of the NLRP3 inflammasome (NLRP3, Casp1 p20, and IL-1β) and pyroptosis executive protein (GSDMD-N) in human endothelial cells treated with lysoPC was analyzed by Western blotting. **I**, **J** Photomicrographs of double-fluorescent staining with propidium iodide (PI) (red) and Hoechst 33,342 (blue) (scale bar represents 100 µm). * p < 0.05 vs. the control group; # p < 0.05 vs. the lysoPC group

To test the cytotoxicity induced by lysoPC in human endothelial cells, we determined cell viability and LDH release. The results showed that lysoPC treatment for 24 h resulted in a significant decrease in cell viability and elevated LDH release in a dose-dependent manner (Fig. 1A, B). Moreover, lysoPC concentration-dependently inhibited the expression of GDF11 in human endothelial cells, suggesting that GDF11 may affect the viability of lysoPC-exposed human endothelial cells (Fig. 1C, D).

As predicted, GDF11 effectively ameliorated cell viability and attenuated LDH release in the lysoPC-treated human endothelial cells (Fig. 1E, F). As described in recent research, lysoPC induces the pyroptosis of human endothelial cells by activating the NLRP3 inflammasome, which is closely related to endothelial cell dysfunction [23, 27]. Then, the impact of GDF11 on the expression of NLRP3, Casp1 p20, IL-1β, and GSDMD-N at the protein level was further investigated. GDF11 pretreatment prevented the activation of the NLRP3 inflammasome and inhibited GSDMD-N expression following lysoPC exposure (Fig. 1G, H). Additionally, we detected a reduced number of PI-positive cells after treatment with GDF11 by Hoechst/PI staining (Fig. 1I, J). Together, these results confirm that GDF11 effectively inhibits lysoPC-induced NLRP3 inflammasome activation and the related occurrence of pyroptosis in human endothelial cells.

### GDF11 promoted re-endothelialization in balloon dilatation-injured rat carotid arteries

**Fig. 2:**
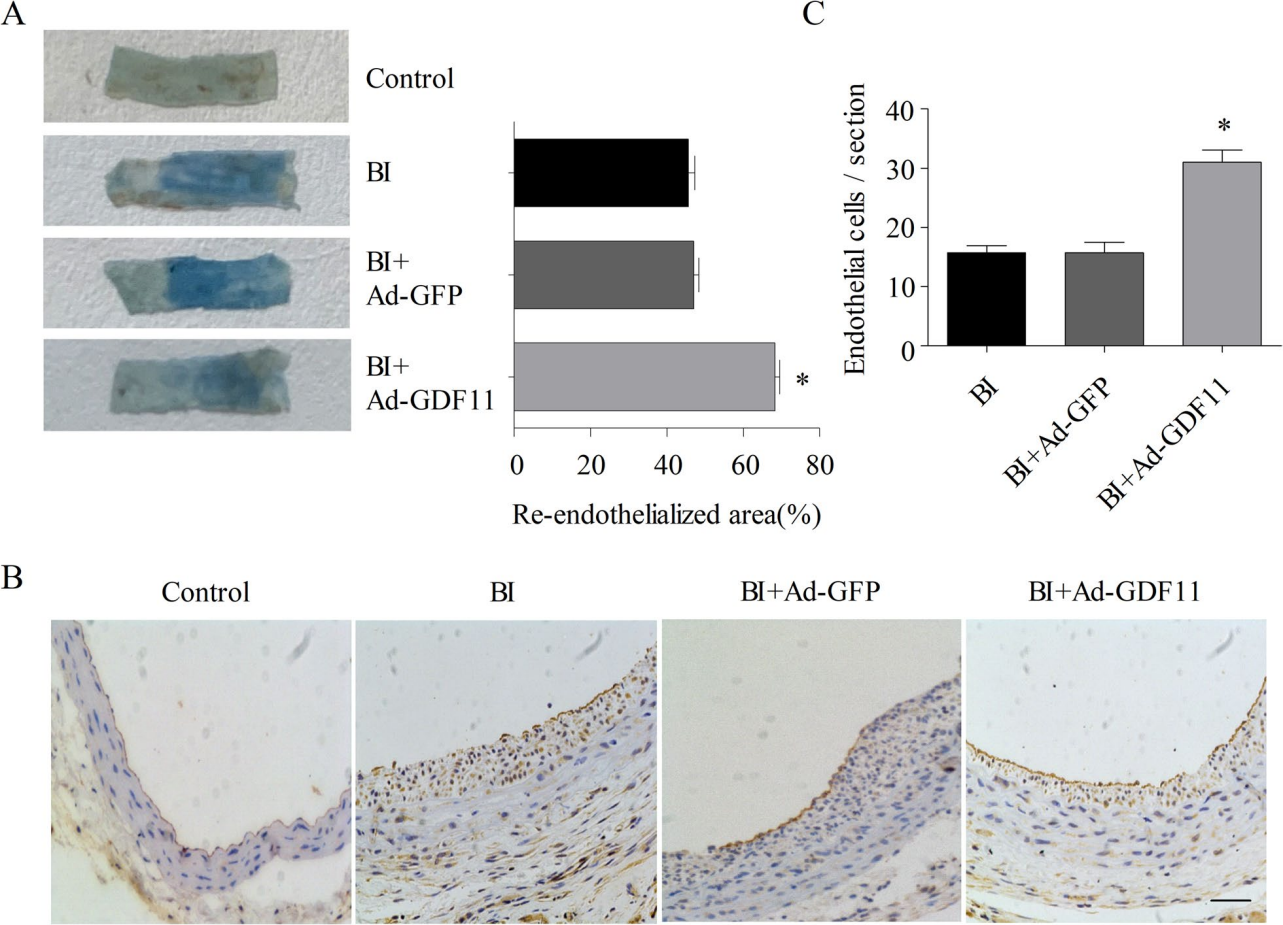
GDF11 promoted re-endothelialization in balloon-dilatation-injured rat carotid arteries. **A** Evans blue-stained carotid arteries at 7 days after vascular injury (representative images); blue staining indicates endothelial denudation. **B** Immunohistochemical staining of CD31 was used to evaluate the re-endothelialization of injured blood vessels in each group (scale bar represents 50 µm). **C** Quantitative result from each group. * p < 0.05 vs. the balloon-injured (BI) group and the BI + Ad–GFP group

LysoPC is closely related to vascular injury and remodeling [28]. To determine whether GDF11 overexpression promotes re-endothelialization in vivo, we used a rat carotid artery balloon injury model. Adenovirus transfection was detected in the carotid arteries by fluorescence microscopy at 3 days after balloon injury and in vivo infection with recombinant adenovirus (Ad) (Additional file 1: Figure S1). Evans blue staining was then used to evaluate re-endothelialization after 7 days of Ad-GDF11 transfection. Our results showed that the re-endothelialization rate of the Ad-GDF11 group was significantly higher than that of the balloon-injured (BI) group (Fig. 2A). Moreover, the number of CD31-positive cells in the Ad-GDF11 group was significantly higher than that in the BI group (Fig. 2B, C).

### GDF11 inhibited NLRP3 inflammasome activation and neointimal hyperplasia in vivo

**Fig. 3:**
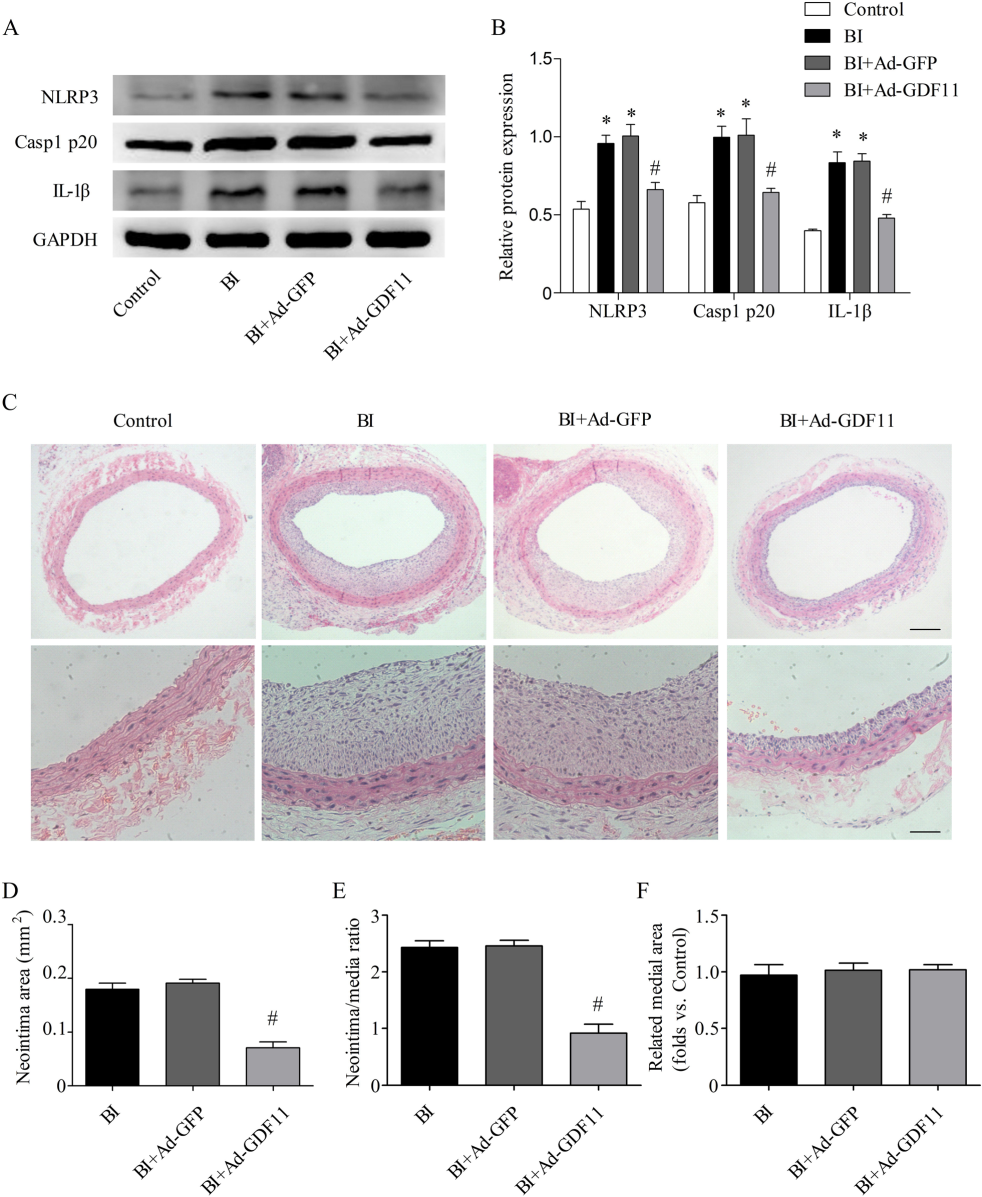
GDF11 inhibited NLRP3 inflammasome activation and neointimal hyperplasia in vivo. **A**, **B** Proteins related to NLRP3 inflammasome activation (NLRP3, Casp1 p20, and IL-1β) were detected by Western blotting. **C** Representative cross-sections with hematoxylin and eosin (HE) staining from different groups (top scale bar represents 200 µm; bottom scale bar represents 50 µm). **D**–**F** Quantitative analysis of the neointimal area, the ratio of neointima to media area and the medial area in different groups. * p < 0.05 vs. the control group; # p < 0.05 vs. the BI group and the BI + Ad–GFP group

To further explore whether GDF11 played an important role through the NLRP3 inflammasome, we evaluated the protein levels of NLRP3, Casp1 p20, and IL-1β at 2 weeks after carotid artery injury. The NLRP3, Casp1 p20, and IL-1β proteins were markedly increased in injured carotid arteries, which were partially reversed by Ad-GDF11 (Fig. 3A, B). Since early re-endothelialization and suppression of the NLRP3 inflammasome can attenuate neointimal hyperplasia, we used HE staining to detect neointimal hyperplasia in each group (Fig. 3C). As expected, the rats with a balloon injury presented significantly more frequently with neointimal hyperplasia in the carotid artery. The BI rats administered Ad-GDF11 showed a significant reduction in the neointimal area compared to the BI rats or rats administered Ad-GFP (Fig. 3D). Similarly, the intimal-to-medial area ratio was significantly reduced in the Ad-GDF11-treated arteries (Fig. 3E). No significant difference in the medial area was observed among all the groups (Fig. 3F). These results demonstrate that GDF11 alleviates neointimal formation in rat carotid arteries after balloon injury.

### GDF11 inhibited NLRP3 inflammasome activation by reducing LOX-1 expression

**Fig. 4:**
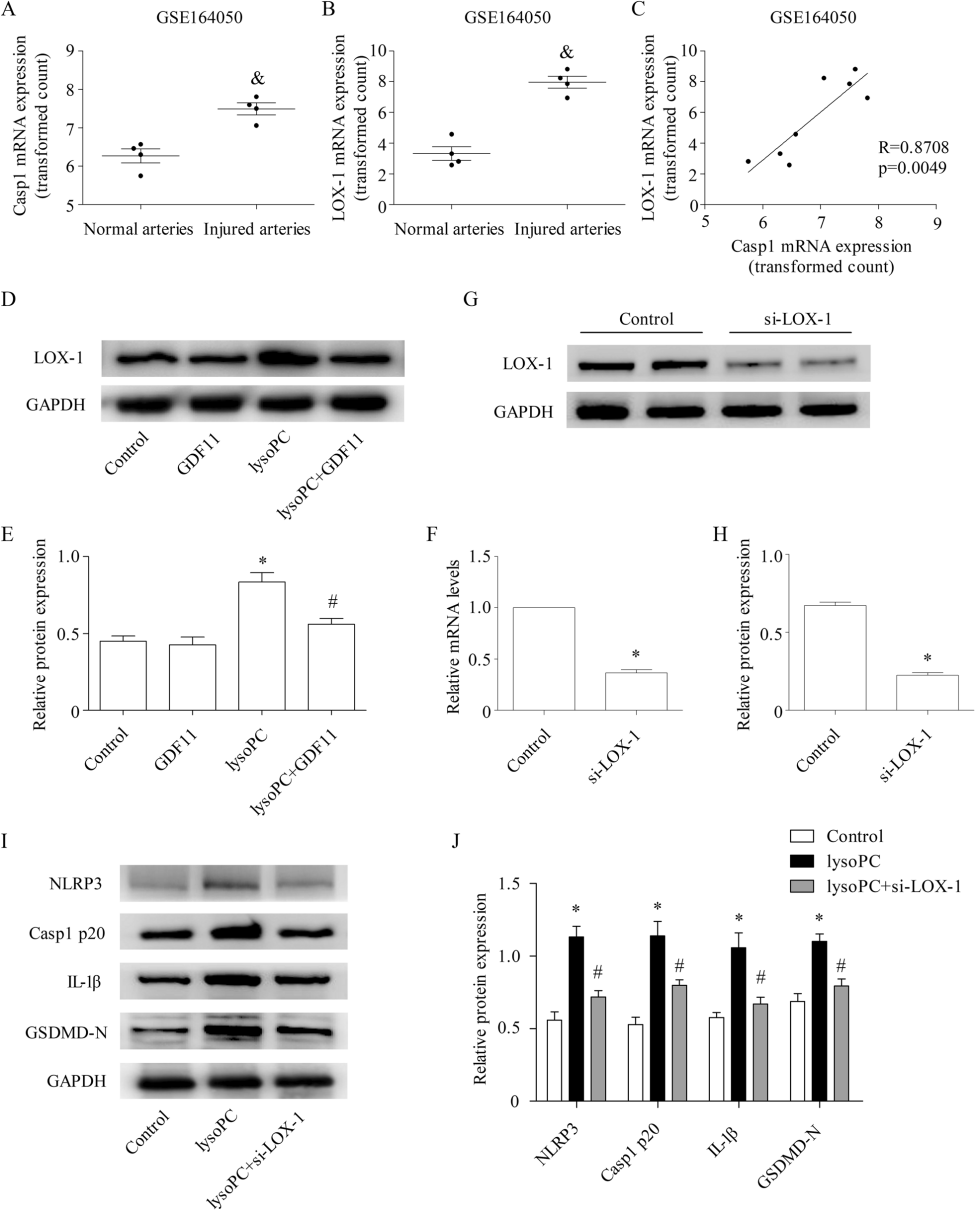
GDF11 inhibited NLRP3 inflammasome activation by reducing LOX-1 expression. **A** Casp1 mRNA levels were higher in injured rat carotid arteries than in normal arteries in cohorts derived from the GEO database (https://www.ncbi.nlm.nih.gov/gds/). **B** The LOX-1 mRNA levels were higher in injured rat carotid arteries than in normal arteries in cohorts derived from the GEO database. **C** There was a positive correlation between LOX-1 and Casp1 transcript levels in the enrolled samples from the GEO datasets. The samples were obtained from injured rat carotid arteries in the GSE164050 dataset. **D**, **E** The effect of GDF11 on the expression of LOX-1 in the human endothelial cells treated with lysoPC was analyzed by Western blotting. **F**–**H** Human endothelial cells were transfected with 50 nM si-LOX-1. At 48 h post-transfection, qPCR and Western blotting were used to detect LOX-1 knockout. **I**, **J** The effect of si-LOX-1 on the expression of NLRP3 inflammasome components (NLRP3, Casp1 p20, and IL-1β) and GSDMD-N in human endothelial cells treated with lysoPC was analyzed by Western blotting. & p < 0.05 vs. the normal arteries; * p < 0.05 vs. the control group; # p < 0.05 vs. the lysoPC group

The results from the public GEO microarray database showed that LOX-1 and Casp-1 were positively correlated with injured carotid arteries in rats (Fig. 4A–C). We also found that GDF11 could inhibit the expression of LOX-1 induced by lysoPC (Fig. 4D, E). To confirm that LOX-1 participated in NLRP3 inflammasome activation in the lysoPC-treated human endothelial cells, we generated si-LOX-1-transfected human endothelial cells and verified them by qPCR and Western blotting (Fig. 4F–H). Our results indicate that si-LOX-1 significantly reversed the lysoPC-induced upregulation of NLRP3, Casp1 p20, IL-1β, and GSDMD-N expression at the protein level (Fig. 4I, J). These results suggest that LOX-1 plays an important role in lysoPC-induced NLRP3 inflammasome activation in human endothelial cells. In addition, GDF11 inhibited NLRP3 inflammasome activation in human endothelial cells by inhibiting the expression of LOX-1.

### Involvement of ER stress in the activation of the NLRP3 inflammasome induced by upregulation of LOX-1 expression

**Fig. 5:**
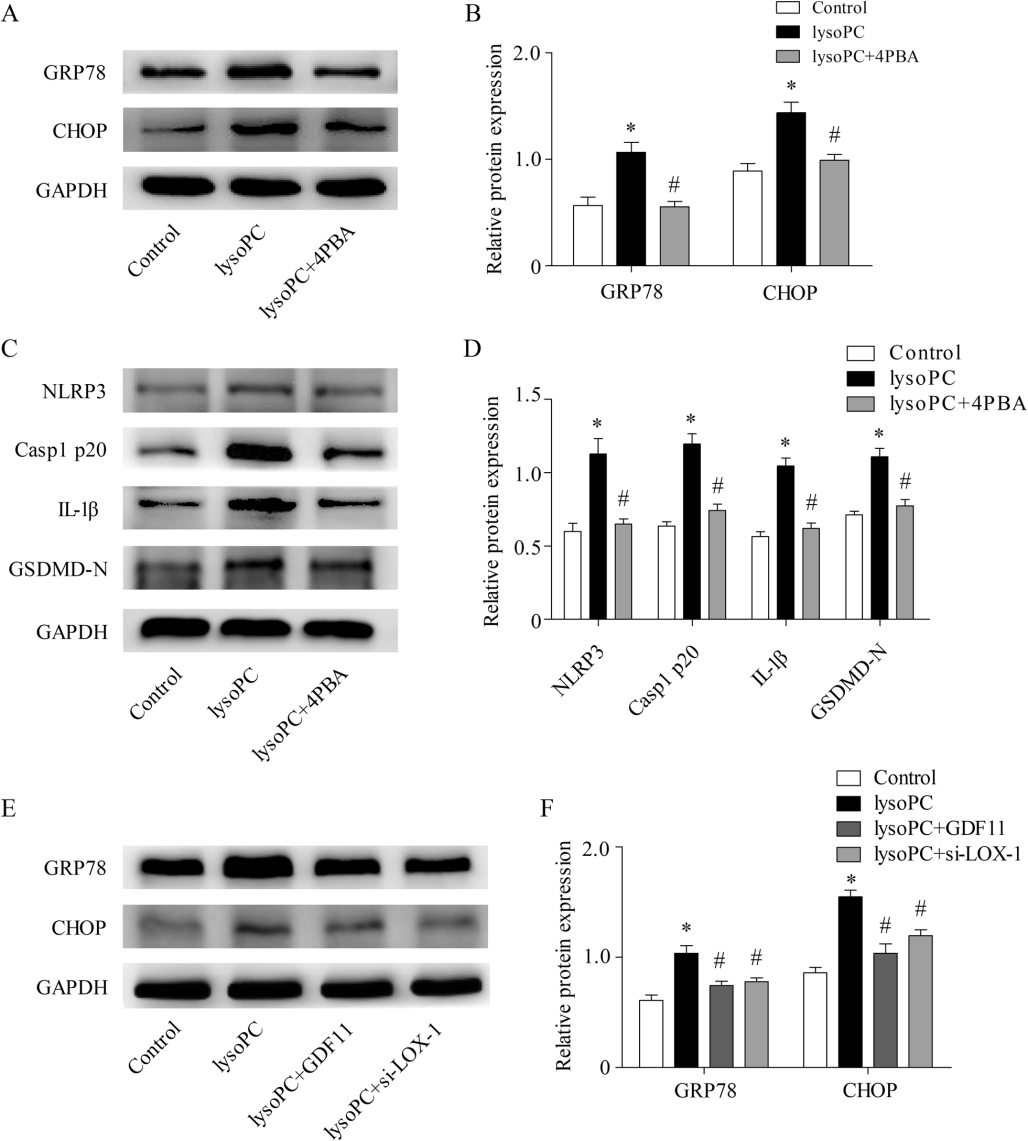
ER stress is involved in the activation of the NLRP3 inflammasome induced by upregulation of LOX-1 expression. **A**, **B** LysoPC increased the expression of proteins related to ER stress (GRP78 and CHOP). The ER stress inhibitor 4PBA (5 mM) inhibited LysoPC-induced ER stress-related proteins. **C**, **D** The ER stress inhibitor 4PBA inhibited NLRP3 inflammasome activation-related proteins (NLRP3, Casp1 p20, and IL-1β) and GSDMD-N expression. **E**, **F** GDF11 and si-LOX-1 pretreatment inhibited lysoPC-induced ER stress in human endothelial cells. * p < 0.05 vs. the control group; # p < 0.05 vs. the lysoPC group

ER stress can regulate NLRP3 inflammasome activation [29, 30]. In human endothelial cells, whether the upregulation of LOX-1 expression regulates NLRP3 inflammasome activation through ER stress remains unclear. Our results showed that lysoPC increased the expression of ER stress markers, such as CHOP and GRP78, compared with that of the control group, while 4-PBA pretreatment decreased the expression of CHOP and GRP78 compared with that of the lysoPC group (Fig. 5A, B). Moreover, in the 4-PBA pretreatment group, the levels of NLRP3, Casp1 p20, IL-1β, and GSDMD-N were significantly reduced (Fig. 5C, D). These results indicate that ER stress participates in the activation of the NLRP3 inflammasome in lysoPC-treated human endothelial cells. In addition, GDF11 treatment and si-LOX inhibited the expression of ER stress markers (Fig. 5E, F). These findings indicate that GDF11 regulates NLRP3 inflammasome activity by inhibiting LOX-1-mediated ER stress.

## Discussion

Previous studies have demonstrated that GDF11 treatment can significantly inhibit the phenotypic changes of vascular smooth muscle cells, improve endothelial function, and attenuate vascular remodeling [7–9]. However, GDF11 regulates a variety of physiological and pathological processes, and multiple signaling pathways are involved in the development of vascular remodeling. Further research is needed to clarify the complex regulatory mechanism of GDF11 in vascular remodeling diseases. In this study, we found that GDF11 alleviated neointimal hyperplasia and promoted rapid re-endothelialization. The protective effect of GDF11 on vascular remodeling is related to the inhibition of NLRP3 inflammasome activation. We also discovered a new and beneficial role for GDF11, as it prevented inflammasome-induced pyroptosis of endothelial cells, and revealed the molecular basis of the GDF11-mediated protection of human endothelial cells. To the best of our knowledge, our results provide the first experimental evidence showing the critical role of GDF11 in the regulation of endothelial NLRP3 inflammasome activation via the negative regulation of LOX-1-dependent ER stress.

LOX-1 is a multiligand receptor that was originally identified as the endothelial ox-LDL receptor. As a risk factor for cardiovascular disease, LOX-1 can be induced by a variety of stimuli, including proinflammatory, oxidative and mechanical stimulation [31]. After vascular injury, the expression of LOX-1 in the injured area increases [32]. In addition, an in vitro culture of regenerated endothelium from BI arteries showed increased expression of modified lipoprotein receptors on the cells [33]. Previous experiments demonstrated that inhibiting LOX-1 could promote re-endothelialization and inhibit restenosis [34]. However, the specific mechanism for this result has yet to be elucidated. Here, we found that the upregulation of LOX-1 expression after arterial injury induced dysfunctional vascular remodeling, which may be related to the activation of the NLRP3 inflammasome. We also found that the upregulation of LOX-1 expression induced the activation of the endothelial NLRP3 inflammasome through ER stress.

Studies have shown that lysoPC can induce the expression of LOX-1 in vascular cells [35, 36]. Moreover, lysoPC can induce the activation of the endothelial NLRP3 inflammasome [22, 23]. Mounting evidence shows that NLRP3 is tightly regulated. Previous investigations demonstrated that excessive ER stress damaged the intracellular microenvironment and eventually affected the activation of the NLRP3 inflammasome [30]. Here, we found that 4-PBA could significantly reduce the ER stress induced by lysoPC while inhibiting human endothelial NLRP3 inflammasome activation. Furthermore, we found that lysoPC could upregulate the expression of LOX-1 in human endothelial cells. When we added si-LOX-1, lysoPC was unable to induce ER stress and human endothelial NLRP3 inflammasome activation.

Some studies have shown that GDF11 can protect endothelial cells by inhibiting apoptosis and senescence [9, 37]. However, the relationship between GDF11 and ER stress has not previously been reported. We found that GDF11 could significantly inhibit the lysoPC-induced expression of ER stress markers. In addition, GDF11 inhibited the expression of LOX-1. Since LOX-1 shows inducible expression, further research is required to determine how the expression of LOX-1 is regulated by GDF11.

Delayed re-endothelialization is related to the dysfunction of endothelial cells [38]. According to the response-to-injury theory, dysfunctional endothelial cells adhering to the damaged vessel wall are related to the mechanism that initiates intimal thickening [39]. Recent studies have found that pyroptosis causes endothelial cell dysfunction [40]. Pyroptosis is also known as inflammatory necrosis of the cell, which is manifested by the continuous expansion of the cell until the cell membrane ruptures and the subsequent release of the cell contents activate a strong inflammatory response [41]. As an important triggering factor and endogenous regulator of cardiovascular inflammation, pyroptosis not only induces the death of endothelial cells but also leads to enhanced endothelial permeability, promotes the expression of endothelial cell adhesion molecules, recruits monocytes and promotes their differentiation into macrophages, and aggravates blood vessel damage [42–44]. Studies have shown that pyroptosis regulated by the NLRP3 inflammasome is closely related to the progression of cardiovascular disease [43, 45]. GDF11 exerts a cardioprotective effect by inhibiting cardiomyocyte pyroptosis induced by the NLRP3 inflammasome [15]. Here, we speculate that GDF11 improves vascular remodeling by inhibiting endothelial cell pyroptosis, as there is also evidence that GDF11 can promote endothelial cell proliferation and angiogenesis and inhibit cell apoptosis [9].

Based on an in vivo experiment, we found that GDF11 promoted re-endothelialization following vascular endothelial injury. Since early endothelialization and inhibition of the NLRP3 inflammasome are closely related to improved restenosis after vascular injury, we found that GDF11 could improve vascular remodeling and inhibit neointima formation through HE staining. The occurrence of restenosis induced by neointima formation is closely related to the proliferation of vascular smooth muscle cells. Recent studies have found that GDF11 can maintain the contractile phenotype of vascular smooth muscle cells under the action of various pathological factors [7, 8, 46]. Therefore, as a protective factor for cardiovascular diseases, GDF11 can play a positive role in improving vascular remodeling.

We acknowledge some limitations that should be mentioned here. First, the rat carotid artery has no obvious atherosclerotic changes before injury, which is somewhat different from clinical balloon angioplasty on diseased blood vessels in human patients. Second, although in vitro studies using HUVEC are common, there are certain differences between venous endothelial cells and arterial endothelial cells. Therefore, further research is needed to verify the protective effect of GDF11 in damaged blood vessel remodeling.

## Conclusion

In summary, and to the best of our knowledge, this study is the first to demonstrate that GDF11 may inhibit endothelial NLRP3 inflammasome activation through the negative regulation of LOX-1-dependent ER stress, thereby reducing endothelial cell pyroptosis and improving endothelial function, which promotes re-endothelialization of damaged blood vessels and thus ameliorates vascular restenosis. These findings reveal a new protective mechanism of GDF11 in remodeling damaged blood vessels and provide a theoretical basis for its clinical application.

## Supplementary Information

**Additional file 1: Figure S1.** Efficiency of adenovirus-mediated gene transfer at 3 days after balloon injury. Representative microphotographs of fluorescence microscopy in different groups. GFP and nuclei with DAPI are labeled by green and blue fluorescence, respectively (scale bar represents 200 µm).

## Data Availability

The datasets used and/or analysed during the current study are available from the corresponding author on reasonable request.

## Abbreviations

GDF11: Growth differentiation factor 11
ER stress: Endoplasmic reticulum stress
LysoPC: Lysophosphatidylcholine
HUVECs: Human umbilical vein endothelial cells
PI: Propidium iodide
si-LOX-1: The small-interfering RNA against LOX-1
HE: Hematoxylin and eosin
Ad: Adenovirus
BI: Balloon-injured
