# Supplementary figures and images for: GDF11 alleviates neointimal hyperplasia in a rat model of artery injury by regulating endothelial NLRP3 inflammasome activation and rapid re-endothelialization
Source: J Transl Med. 2022 Jan 15;20:28. doi: 10.1186/s12967-022-03229-6 (PMC8760779; doi:10.1186/s12967-022-03229-6)

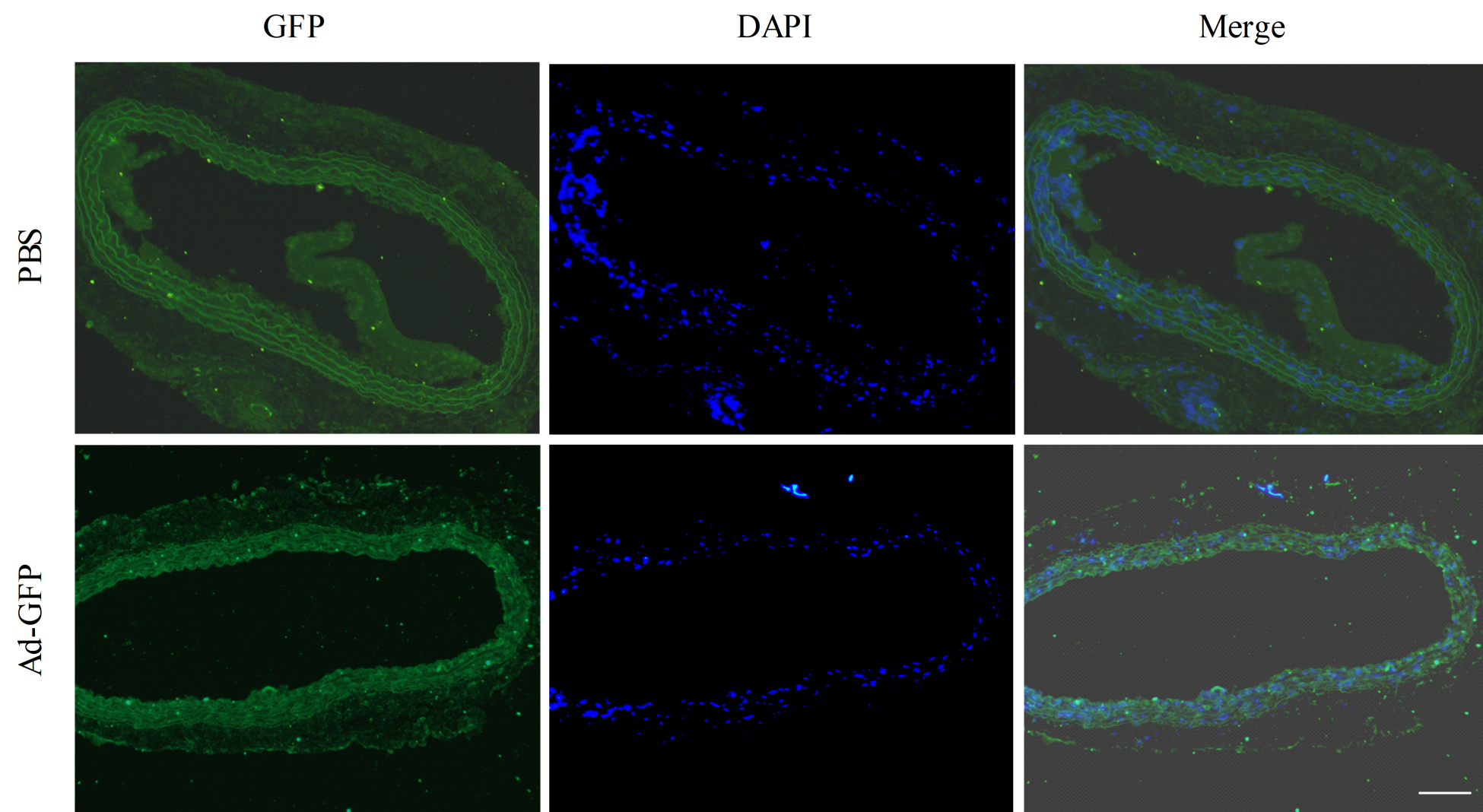

Supplement: Supplementary file 1 — Additional file 1: Figure S1. Efficiency of adenovirus-mediated gene transfer at 3 days after balloon injury. Representative microphotographs of fluorescence microscopy in different groups. GFP and nuclei with DAPI are labeled by green and blue fluorescence, respectively (scale bar represents 200 µm). [file 12967_2022_3229_MOESM1_ESM.pdf]
